# Sex-specific neural responses to smartphone cues in young adults

**DOI:** 10.1186/s13293-026-00835-7

**Published:** 2026-01-31

**Authors:** Nadine D. Wolf, Mike M. Schmitgen, Gudrun M. Henemann, Sophie Haage, Patrick Bach, Julian Koenig, Robert Christian Wolf

**Affiliations:** 1 Department of General Psychiatry, Center for Psychosocial Medicine, Heidelberg University, Voßstraße 4, 69115 Heidelberg, Germany; 2 DZPG, German Center for Mental Health, Partner Site Mannheim, Heidelberg, Ulm (ZIHUb), Heidelberg, Germany; 3 Department of Addictive Behavior and Addiction Medicine, Central Institute of Mental Health, Mannheim, Germany; 4 Faculty of Medicine and University Hospital Cologne, Department of Child and Adolescent Psychiatry, Psychosomatics and Psychotherapy, University of Cologne, Robert-Koch-Strasse 10 (Building 53), 50931 Cologne, Germany

**Keywords:** FMRI, Smartphone use, Cue reactivity, Sex differences

## Abstract

Problematic smartphone use has been associated with altered reward and executive control network activity, yet potential sex differences in the underlying neural mechanisms remain insufficiently understood. We investigated sex-specific neural correlates of smartphone cue reactivity (CR) in 69 healthy young adult smartphone users (age range 18–30 years, female/male *n* = 45/24). Participants completed the Smartphone Addiction Inventory (SPAI) and underwent functional MRI during a smartphone CR paradigm. In addition, resting-state data were acquired to ensure that neural differences between female and male participants could be attributed to the CR paradigm rather than to sex differences in intrinsic neural activity. Whole-brain analyses revealed stronger activation in males compared to females in response to the presentation of smartphone cues within the right middle frontal gyrus (MFG), thalamus, cortical sensorimotor, parietal and occipital regions, whereas females showed no suprathreshold clusters compared to males. No overlap with resting-state amplitude of low-frequency fluctuation maps was observed with CR results, confirming task specificity. In males, right MFG correlated positively with SPAI-I total score, craving, and sleep interference scores, while in females, right parietal cortex activity correlated negatively with SPAI-I total score, daily life interference, and craving. Complementary cross-modal analyses showed that CR-related activation patterns were associated with several cortical excitatory and inhibitory neuronal and cellular markers, revealing subtle sex differences. These findings suggest sex-specific frontoparietal mechanisms underlying smartphone CR and highlight neurochemical pathways potentially linking excessive smartphone use to differential motivational and cognitive control processes in males compared to females.

**Supplementary Information:**

The online version contains supplementary material available at 10.1186/s13293-026-00835-7.

## Highlights

Males showed stronger smartphone cue-reactivity across frontoparietal, thalamic, sensorimotor, and occipital regions compared with females.

Right middle frontal gyrus activation in males correlated positively with the extent of smartphone use, craving, and sleep interference.

In females, right parietal cortex activation correlated negatively with the smartphone use levels, craving, and daily life interference.

Cross-modal analyses pointed to shared excitatory and inhibitory neurochemical substrates, while revealing subtle sex-specific differences in how these neural markers relate to behavior.

**Supplementary Information:**

The online version contains supplementary material available at 10.1186/s13293-026-00835-7.

## Abstract (plain language summary)

People use smartphones constantly, and for some individuals this can become difficult to control. One reason may be that certain smartphone-related cues, such as notifications or images of phones—trigger strong reactions in the brain. However, it is not well understood whether these reactions differ between females and males. In this study, we examined how the brains of 69 young adults respond to smartphone cues while lying in an MRI scanner. We also measured their usual smartphone use and collected resting-state brain data to make sure any differences we observed were truly related to the cue task.

We found clear sex differences. When viewing smartphone cues, males showed stronger activity in brain regions involved in attention, decision-making, sensory processing, and visual perception, particularly in a frontal brain area linked to control of behavior. In contrast, females did not show stronger activity than males in any brain region, but instead showed a different pattern: activity in a parietal region was lower in those with more problematic smartphone use. These brain responses were specific to the cue task and not present in resting-state data. We also examined how these patterns relate to underlying brain chemistry. The activation seen during cue reactivity was connected to several markers of excitatory and inhibitory neural signaling, with subtle differences between females and males. Overall, our findings suggest that males and females engage different brain mechanisms when reacting to smartphone cues, which may help explain why excessive smartphone use affects them differently. These insights may support future strategies tailored to sex-specific motivational and self-control processes.

**Supplementary Information:**

The online version contains supplementary material available at 10.1186/s13293-026-00835-7.

## Introduction

Smartphones have become integral to daily life, enabling constant communication, entertainment, and access to information. However, their excessive use has raised concerns regarding potential maladaptive behavioral and neurobiological consequences [1–3]. Problematic smartphone use (PSU) is increasingly conceptualized within the framework of behavioral addictions, sharing clinical and neurocognitive features with substance use and other technology-related disorders, such as Internet gaming disorder (IGD) [4–6]. Individuals with high PSU scores often report impaired self-control, compulsive checking behavior, craving-like urges, and interference with daily functioning, i.e. symptoms resembling those observed in established addictive behaviors [3].

Recent epidemiological and behavioral studies indicate that PSU is highly prevalent and exhibits clear sex-specific patterns. Meta-analytic data estimate a global prevalence of approximately 37%, with males showing faster growth over time despite initially lower rates [7]. In adolescents, “dual-problem” users (i.e. those presenting with both problematic Internet and smartphone use) are more often male, while females are more likely to show problematic smartphone use with heightened impulsivity and aggression [8]. Among college students, males’ smartphone addiction is associated with gaming, anxiety, and poor sleep quality, whereas females’ is linked to multimedia and social networking use, depression, anxiety, and disrupted sleep [9]. Bedtime smartphone use has been specifically linked to higher anxiety severity, with worry-related symptoms showing the strongest associations and greater effects in females [10]. Protective factors, such as parental awareness in adolescents and social support in adults, also exhibit sex-specific influences [11], underscoring the need to account for sex-related factors in understanding the development and maintenance of PSU [9, 12–14].

Paralleling the epidemiological findings, the neuroimaging database on the neural mechanisms underlying PSU has been growing steadily in the past decade [1, 3, 15, 16]. At the neural level, converging evidence suggests that cue-reactivity (CR) paradigms provide valuable insights into the mechanisms of craving and habit formation in the study of PSU. In substance-related and behavioral addictions, exposure to disorder-relevant cues elicits activity in frontostriatal, parietal, and limbic networks implicated in motivational salience and executive regulation [6, 17]. In PSU, this paradigm has been recently adapted to present smartphone-related stimuli, distinguishing between active (screen-on) and inactive (screen-off) devices, which elicit both hyper- and hypoactivation across frontoparietal, salience, and motor networks [18–20]. Longitudinal studies indicate that neural responses, particularly in reward-processing regions such as the anterior cingulate cortex and striatum, are dynamically modulated by short-term smartphone restriction, and that these responses are associated with symptom severity, including compulsive use and craving [19].

Despite the steadily growing neuroimaging-based evidence on PSU, most studies have focused on mixed-sex samples or statistically controlled for sex, without explicitly examining sex-specific neural mechanisms [3]. This is particularly surprising given that epidemiological and behavioral research consistently demonstrates robust sex differences in smartphone-related problems, while neural correlates underlying these remain largely unexplored. This observation is even more surprising since sex differences in the neural correlates of reward processing, impulsivity, and emotion regulation are well established [21–24]. Females and males differ in dopaminergic and serotonergic system function, in cortical excitability patterns, and in the neuroendocrine modulation of frontolimbic circuits [25, 26]. Such differences have been linked to variations in susceptibility, symptom expression, and relapse risk in addictive and compulsive behaviors [27–29].

Yet, despite the rapid growth of smartphone use worldwide and consistent reports of sex-related behavioral differences in PSU, such as higher self-reported social and emotional use motives in females and stronger performance or gaming motives in males, neural sex differences in smartphone CR remain largely unexplored. Furthermore, the neurochemical basis of putative sex-specific effects in smartphone CR has not been investigated in vivo. Understanding whether neural activation patterns during smartphone CR differ by sex, and if they align with specific receptor systems may elucidate the biological pathways that are tightly coupled to excessive smartphone engagement. To fill these gaps, in the present study we aimed to characterize sex-specific neural correlates of smartphone CR in healthy young adults using functional magnetic resonance imaging (fMRI). Participants were exposed to smartphone-related and neutral visual stimuli while undergoing fMRI, and psychometric data on smartphone use behavior were assessed. To ensure that observed effects were task-specific and not confounded by intrinsic resting-state activity, we additionally analyzed resting-state amplitude of low-frequency fluctuations (ALFF) [30]. Finally, we applied cross-modal correlations with PET/SPECT-derived receptor and cellular marker maps using the JuSpace toolbox [31] to explore underlying neurochemical signatures. We hypothesized that males and females would exhibit distinct prefrontal and parietal activation patterns during smartphone CR and that these differences would relate to specific dimensions of smartphone use behavior and to neurochemical systems implicated in cognitive control, attention and reward processing.

## Materials and methods

### Participants

In this study, we included cross-sectional data of 69 young adult smartphone users from two projects investigating neural mechanisms of CR in smartphone users (29 subjects from [20] and 40 participants from [19] [MRI-data from the first MRI-session]; 21 and 24 females, respectively). Inclusion criteria were as described in the two studies mentioned above and by [30]: sufficient German language skills, right-handedness, age 18–30 years, no general contraindications for MRI or self-reported neurological or mental illness, no IGD, as indicated by cut-off scores of < 6 on the short form of Internet Gaming Disorder Scale (IGDS-sf) [32] and only participants with sufficient MRI data quality and without missing values in psychometry were selected for the present study.

Written informed consent was given by all participants prior to inclusion in our study. The Ethics Committee of the Medical Faculty at Heidelberg University, Germany approved our study, which was carried out in compliance with the Declaration of Helsinki. All participants received monetary compensation for their participation in our study.

### Procedure

#### Psychometric assessment

Participants completed the short version of the Smartphone Addiction Scale (SAS-SV) [33] and the short form of the Internet Gaming Disorder Scale (IGDS-sf) [34]. The SAS-SV is an instrument that captures the magnitude of physical, psychological, and social problems associated with smartphone use. It comprises 10 items with a maximum score of 60. The IGDS-sf comprises 9 items and it captures psychological and social problems caused by computer gaming in a binary manner (yes/no questions) within the last year and was used to exclude participants with IGD. At inclusion [19, 20], all participants completed the Smartphone Addiction Inventory (SPAI) [35] to evaluate the overall quantity of smartphone use and associated smartphone-use dimensions. In this study, a five-factor model (SPAI-I [36]), was used (see Table 1 for demographic and psychometric data). Further psychometric data included the Beck Depression Inventory (BDI-II) [37].

**Table 1:** Demographics and psychometric scores by group and differences between groups

	Full sample *n* = 69 (mean)	SD	Min-Max	Female *n* = 45 (mean)	SD	Min-Max	Male *n* = 24 (mean)	SD	Min-Max	Statistic (df)	*p*_female vs. male_	Effect size (Cohen’s *d*)
Age	22.7	2.6	18–29	22.5	2.6	18–29	23.1	2.6	18–29	−0.91 (67)^a^	0.367	0.23
BDI total	5.5	5.8	0–24	6.8	6.2	0–24	3.1	4.0	0–17	734.5^b^	0.014	−0.65
SPAI-I total	42.8	14.2	25–86	44.5	15.3	25–86	39.5	11.3	26–65	631.5^b^	0.251	−0.29
SPAI-I time spent	8.2	3.1	4–16	8.4	3.2	4–16	7.8	2.8	5–14	598^b^	0.464	−0.19
SPAI-I compulsivity	5.6	2.0	4–12	5.9	2.2	4–12	5.1	1.4	4–8	623^b^	0.272	−0.28
SPAI-I daily life interference	12.5	4.5	8–29	13.0	5.0	8–29	11.7	3.1	8–21	583.5^b^	0.586	−0.14
SPAI-I craving	11.0	4.1	5–20	11.4	4.2	5–20	10.1	3.9	5–18	1.26 (67)^a^	0.213	0.32
SPAI-I sleep interference	5.5	2.5	3–11	5.9	2.6	3–11	4.9	2.3	3–11	663^b^	0.112	−0.41

*SD* standard deviation, *Min* minimum value, *Max* maximum value; *BDI* Beck-Depression-Inventory, *SPAI-I* Five-factor model of the Smartphone Addiction Inventory

^a^*t*-value, ^b^Wilcoxon *W*

##### MRI data acquisition

Whole-brain MRI-images were acquired using a 3 T SIEMENS MAGNETOM TIM Trio (dataset from Schmitgen et al. 2020) and a 3 T SIEMENS MAGNETOM Prisma Fit MRI-scanner (SIEMENS, Erlangen; dataset from Schmitgen et al., 2025) equipped with a 32-channel head coil in a darkened room. The head of the participants was fixed using foam cushions to minimize head motion. The scanner protocol included four [20] or five [19] functional EPI-BOLD measurements including (in this order) a resting state scan, the cue-reactivity experiment, two [20] or three [19] further experimental paradigms, and a structural scan.

Parameters of the sequences used for data analysis here were as follows:

- CR experiment from [20]: 293 whole-brain EPI volumes were recorded in transverse (axial) orientation with repetition time (TR) = 2.20 s, echo time (TE) = 29 ms, field of view (fov) = 192 × 192 × 143 mm, flip angle = 85°, voxel size = 3 × 3 × 3 mm, 36 slices, distance factor between slices = 33%.
- CR experiment from [19]: 293 whole brain EPI volumes were recorded in transversal orientation with TR = 2.22 s, TE = 29 ms, fov = 192 × 192 × 131 mm, flip angle = 85°, voxel size = 3 × 3 × 3 mm, 33 slices, distance factor between slices = 33%.

The resting state parameters and instructions from the first study sample are described in detail in [30]. Parameters of the resting state sequence from the second study sample were as follows: participants were instructed to keep their eyes closed, not to fall asleep, and not to think about anything specific. 200 whole brain echo planar imaging (EPI) volumes were recorded in a transverse (axial) orientation, repetition time = 2020 ms, echo time = 29 ms, field of view = 192 mm, flip angle = 90°, voxel size = 3 × 3 × 3 mm, 33 slices recorded in sequential ascending order, distance factor between slices = 1 mm.

##### CR-task

**Fig. 1:**
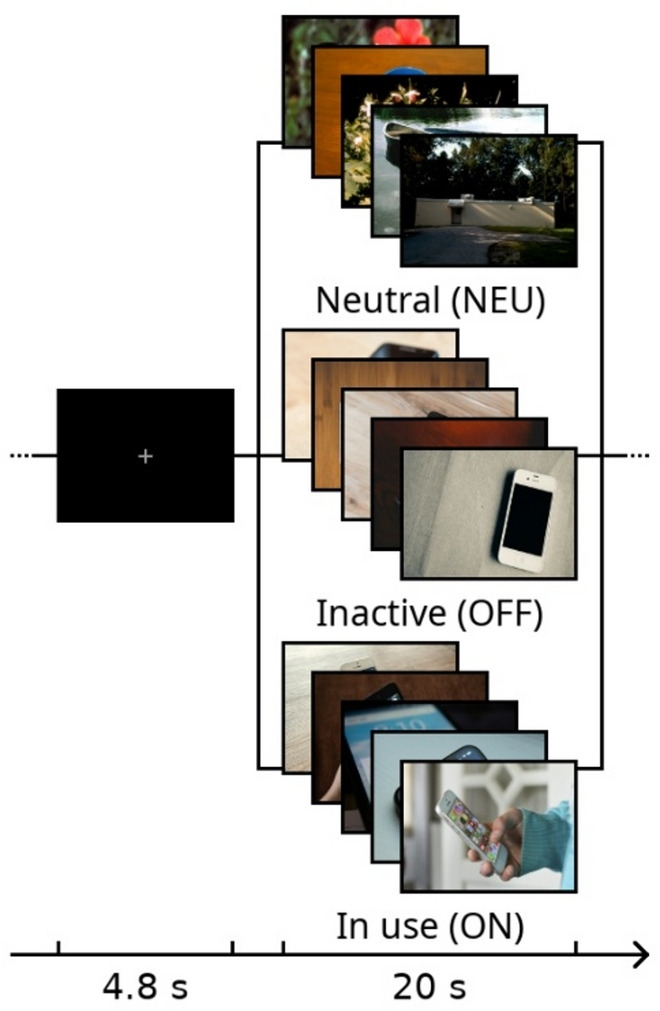
Schematic overview of the CR-task. This figure was created using GIMP (https://www.gimp.org/; last visited 10/14/2025)

In both studies [19, 20], the modified block-design CR-task as described by [20], based on the CR-task by Beck and coworkers [38]. In brief, during the task, blocks of five pictures were presented for 20 s and a fixation cross (ITI; 25 blocks, one before the start of the experiment and 24 between blocks of pictures) was presented for 4.8 s between the blocks of pictures. The blocks of pictures either contained neutral images (NEU; 12 blocks), inactive smartphones (OFF; six blocks), or smartphones in use (ON; six blocks; see Fig. 1). Block order and pictures within blocks were pseudorandomized between participants.

### Data analysis

#### Psychometric data

Age, BDI, and SPAI-I total score and its five factors were tested for differences between female and male participants via two-samples t-tests or non-parametric methods, if the data was not normally distributed (i.e. two-samples Wilcoxon test; see Table 1 for demographics, psychometric scores, and test results).

#### FMRI

##### FMRI data preprocessing

Task-based fMRI images were preprocessed using the Data Processing Assistant for resting state fMRI (DPABI/DPARSF) [39]. In accordance with our previous studies [19, 20], preprocessing included slice timing, realignment, reorientation of functional and structural data, automated mask-creation, brain extraction, coregistration of the T1 image to functional images, SPM new segment and DARTEL using affine regularization for European participants, normalization by DARTEL to a voxel-size of 3 × 3 × 3 mm, and smoothing using a FWHM of 9 × 9 × 9 mm. Head movement > 3 mm or 3° was defined as exclusion criteria.

To complement the findings from the CR-task, particularly to ensure that these findings are specific to the CR paradigm, and not to sex-specific difference in intrinsic neural activity, resting state data were analyzed in terms of amplitude of low-frequency fluctuations (ALFF). Preprocessing of the resting state data comprised the same parameters as for the task-based data and additionally included band-pass filtering at 0.01 to 0.1 Hz.

##### FMRI data analysis, CR-paradigm: 1 st level contrasts

Statistical fMRI data analysis was conducted using SPM12 (https://www.fil.ion.ucl.ac.uk/spm/; last visited 10/14/2025). As in our previous studies [19, 20], 1 st level models (block design) were set up using the following regressors:

###### PHONE vs. NEU model

ITI, blocks of pictures showing ON or OFF (PHONE), blocks of pictures showing NEU, and six movement parameters.

###### ON vs. OFF model

ITI, blocks of pictures showing NEU, blocks of pictures showing OFF, blocks of pictures showing ON, and six movement parameters.

##### FMRI data analysis, CR-paradigm: group inference

As the primary aim of this study was to delineate sex-specific differences in CR-related neural activity, females and males were grouped irrespective of their self-reported smartphone use levels. SPM two-samples t-tests for contrasts of PHONE > NEU and ON > OFF for female > male and male > female were set up to visualize neural sex-differences of *p* < 0.001 uncorrected for height, followed by an empirically determined cluster extent threshold based on Gaussian random field theory as implemented in SPM, using image smoothness estimates from the residual fields to determine the minimum cluster size corresponding to a cluster-level significance of *p* < 0.05. This threshold was defined a priori and applied consistently across contrasts to control the family-wise error rate at the cluster level while retaining sensitivity for exploratory whole-brain analyses [40, 41]. Age and MRI-scanner were used as covariates of no interest in these models. Statistical maps from the task-based data were masked with the respective resulting ALFF maps to test for task-specificity of the CR-related findings.

A similar group inference procedure was applied for ALFF data (using framewise displacement measures according to Power and colleagues [42] as an additional covariate).

##### Correlations between region of interest task-fMRI activation and psychometric scores

Further, raw activation within brain regions detected in the group comparisons (PHONE > NEU female vs. male and ON > OFF female vs. male; left and right hemisphere separately for close to midline structures) was extracted using MarsBaR (version 0.45 [43] and used for Spearman correlations with psychometric scores in R (version 4.5.1; https://www.r-project.org/; last visited 10/14/2025). Anatomical masks were created using the Neuromorphometrics atlas implemented in SPM12.

##### Cross-modal correlations between task-fMRI activation patterns and PET/SPECT-derived receptor maps and cellular markers

Cross-modal Spearman correlations of activation patterns of PHONE > NEU female vs. male, ON > OFF female vs. male, PHONE > NEU female and male together, and ON > OFF female and male together with PET/Neurotransmitter templates and cellular markers (all 71 templates available and implemented in JuSpace at the time of data analysis) were calculated via JuSpace toolbox (version 2.0; https://github.com/juryxy/JuSpace; last visited 10/14/2025) [31]. The JuSpace toolbox allows for cross-modal correlations between MRI-imaging data and PET/SPECT-derived receptor maps and cellular markers. In JuSpace, option 5 (“converts each image in set 1 to z-scores relative to images in set 2”) was chosen to calculate differences of Spearman correlations between female and male participants. For completeness, this procedure was also computed across the entire sample, i.e. independently of the participant’s sex, using JuSpace’s option 3 (“uses mean per region from set 1”). For computation of exact *p*-values 10,000 permutations were used. For these analyses a nominal significance level of *p* < 0.05 was chosen and FDR-correction was applied.

## Results

### Demographics and psychometric scores

SPAI-I total and SPAI-I subscores did not significantly differ between sexes. BDI showed a significant difference between female and male participants (*p* = 0.014; see Table 1 for further details).

### Between-group differences in brain activity

**Fig. 2:**
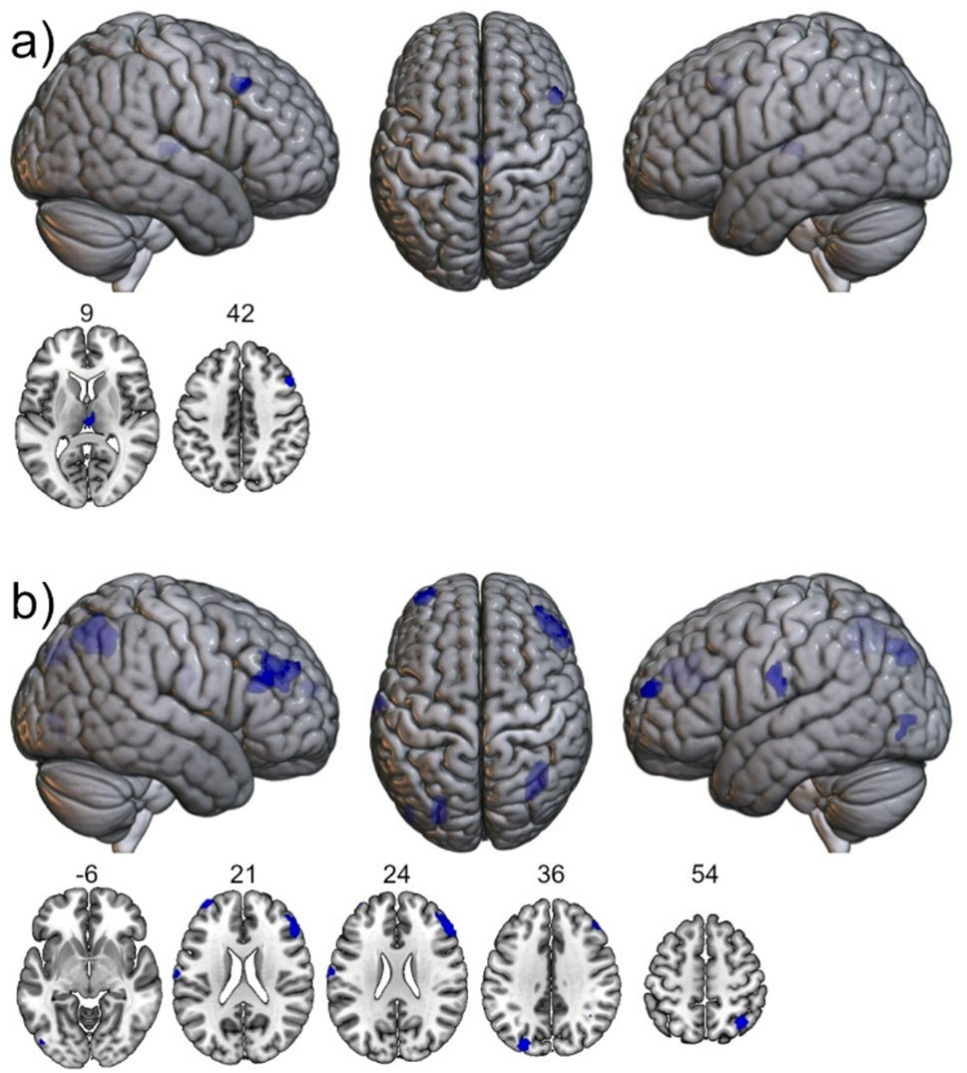
Localizations of CR-related brain activity differences between males and females. (**a**) PHONE > NEU male > female at *p* < 0.001, *k* = 14. Top: renderings onto the mni152 template from right, superior, and left view. Bottom: axial slices at cluster maxima in the z-plane. Numbers show MNI-coordinates in the z-plane. (**b**) ON > OFF male > female at *p* < 0.001, *k* = 13. Top: renderings onto the mni152 template from right, superior, and left view. Bottom: axial slices at cluster maxima in the z-plane. Numbers show MNI-coordinates in the z-plane. This figure was created using MRIcroGL (https://www.nitrc.org/projects/mricrogl; last visited 10/14/2025) and GIM

The SPM two-samples t-test model of the contrast PHONE > NEU male > female showed increased activation in right middle frontal gyrus and right thalamus at *p* < 0.001 (uncorrected), k = 14 voxels (see Fig. 2; Table 2). The contrast PHONE > NEU female > male did not show any significant clusters. The contrast ON > OFF male > female revealed greater activation in right angular gyrus, left postcentral gyrus, left middle occipital gyrus, left and right middle frontal gyrus, and left inferior occipital gyrus at *p* < 0.001 (uncorrected), k = 13 voxels (see Fig. 2; Table 2). ON > OFF female > male did not show any significant clusters.

**Table 2:** Differences of brain activation between groups

Brain area	Cluster size *k* (voxel)	*T* value (peak voxel)	Peak voxel coordinates (MNI)		
			*x*	*y*	*z*
*PHONE > NEU male > female; k = 14*					
R middle frontal gyrus	20	4.46	48	21	42
R thalamus	25	4.20	3	−18	9
*PHONE > NEU female > male; k = 14*					
-	-	-	-	-	-
*ON > OFF male > female; k = 13*					
R angular gyrus	124	4.23	36	−60	54
L postcentral gyrus	27	4.19	−66	−9	24
L middle occipital gyrus	56	4.18	−27	−81	36
L middle frontal gyrus	29	4.10	−36	60	21
R middle frontal gyrus	127	3.96	51	30	21
L inferior occipital gyrus	16	3.54	−48	−81	−6
*ON > OFF female > male; k = 13*					
-	-	-	-	-	-

Masking these results with the respective ALFF-maps derived from the resting state data showed no spatial overlap with clusters exhibiting between-group differences (see Table S1 for cluster localizations in the ALFF-maps).

### Associations between psychometric scores and brain activation

**Fig. 3:**
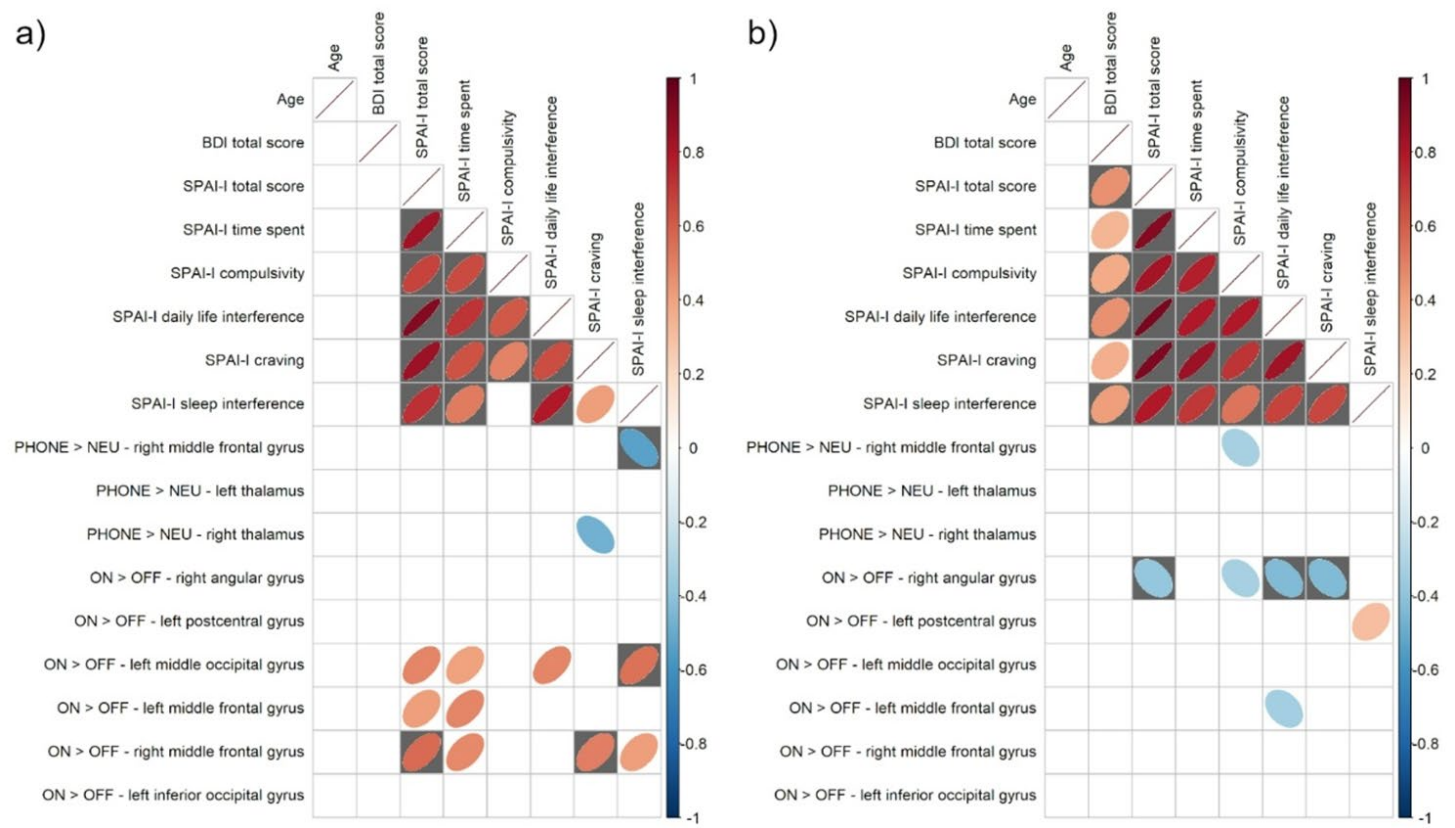
Correlation matrices of Spearman rank correlations between psychometric data and task-based brain activity within regions of interest based on group differences. (**a**) Male participants, (**b**) female participants. Ellipses depict significant correlations at the *p* < 0.05 level, tests surviving FDR-correction are highlighted in gray color. This figure was created using R and GIMP

In the contrast of PHONE > NEU, male participants showed a significant (FDR-corrected) negative correlation between SPAI-I sleep interference and activation of the right middle frontal gyrus. In the contrast of ON > OFF, male participants showed significant (FDR-corrected) positive correlations between SPAI-I total score and activation of the right middle frontal gyrus, between SPAI-I craving and the right middle frontal gyrus, and between SPAI-I sleep interference and the left middle occipital gyrus (see Fig. 3 for details).

In the contrast of ON > OFF, female participants showed significant negative correlations of activation of the right angular gyrus with SPAI-I total score, SPAI-I daily life interference, and SPAI-I craving (see Fig. 3 for details).

In the contrast of ON > OFF, female and male participants taken together showed a significant (FDR-corrected) negative correlation between SPAI-I compulsivity and activation of the right angular gyrus (see Figure S1 for details).

### Cross-modal correlations between functional CR and PET/SPECT-derived receptor maps and cellular markers

#### Between-group differences

**Fig. 4:**
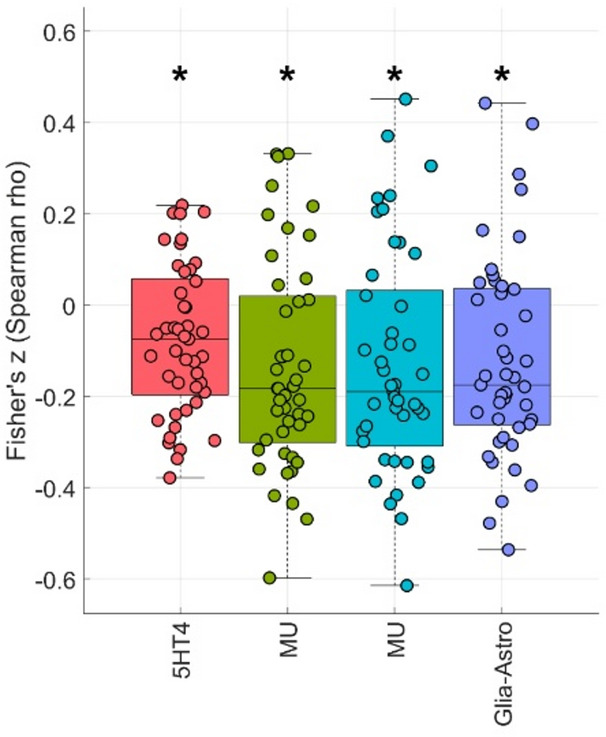
Differences of cross-modal Spearman correlations between brain activation and receptor probability maps/cellular markers. PHONE > NEU female vs. male, *exact *p* < 0.05, none of the in-total 71 correlations survived FDR-correction. This figure was created using JuSpace and GIMP

For the contrast PHONE > NEU female vs. male 5HT4, MU, and Glia-Astro maps showed differences of cross-modal correlations between female and male participants (*p* < 0.05), yet none of the in-total 71 correlations survived FDR-correction (see Fig. 4 and Figure S3). For the contrast ON > OFF female vs. male none of the maps showed differences of cross-modal correlations between female and male participants.

#### Associations within the combined sample

For the contrast PHONE > NEU female and male together 5HT4, CB1, CBF, COX1, D2, DAT, NAT, NMDA, SERT, VAChT, VMAT2, mGluR5, neuron-Ex2-Granule-L34, neuron-Ex4-SubcortProject-L4, neuron-Ex5-SubcortProject-L456, neuron-Ex7-CorticothalProject, neuron-In4-RELN-NDNF-L13, and, neuron-In7-SST-CALB-NPY-L56 maps showed cross-modal correlations (*p* = 0.05), whereas CB1, DAT, SERT, VAChT, VMAT2, neuron-Ex4-SubcortProject-L4, neuron-Ex5-SubcortProject-L456, and neuron-In7-SST-CALB-NPY-L56 maps survived FDR-correction (see Figure S2 and Figure S4).

For the contrast ON > OFF female and male together 5HT6, A4B2, CB1, CMRglu, D1, D2, DAT, FDOPA, GABAa5, HDAC, KOR, KappaOp, M1, MOR, MU, NAT, NMDA, SERT, SV2A, VAChT, VMAT2, mGluR5, Glia-Endo, Glia-Oligo, neuron-Ex8-CorticothalProject-L6, and neuron-In6-PVALVB-L45 maps showed significant cross-modal correlations (*p* = 0.05), whereas HDAC, one MU, one SERT, and neuron-Ex8-CorticothalProject-L6 maps did not survive FDR-correction (see Figure S2 and Figure S4).

## Discussion

In the present study, we examined sex-specific neural correlates of smartphone CR in healthy young adults using fMRI, complemented by resting-state ALFF analyses and cross-modal correlations with PET/SPECT-derived neurochemical maps. We found that males, compared to females, exhibited greater activation in prefrontal, parietal, and occipital regions during smartphone-related stimulation. Brain–behavior correlations revealed opposing associations between smartphone use dimensions and neural activation in males and females, particularly within the middle frontal and parietal cortices. Exploratory cross-modal analyses revealed significant associations between smartphone CR and multiple excitatory and inhibitory mechanisms neurotransmitter and cellular systems, although in these analyses, robust sex-specific differences were not detected.

The present findings highlight a distinct pattern of male-specific brain activation in response to smartphone-related cues, emphasizing the central involvement of the frontal cortex in modulating cue reactivity [6, 20, 38, 44–46]. In the PHONE > NEU contrast, men exhibited increased activation in the right middle frontal gyrus (MFG) and right thalamus, regions critically implicated in executive control, attention allocation, and motivational salience processing [47–49]. The frontal engagement is consistent with evidence highlighting the prefrontal cortex’s key role in CR in substance-use disorders [50]. In smartphone CR, the present data suggest that male participants engage higher-order cognitive control networks when exposed to smartphone cues. The repeated involvement of the MFG across both contrasts underscores the robustness of frontal contributions to smartphone cue processing in men. Importantly, this frontal activation pattern was not observed in females, indicating potential sex differences in the neural substrates underlying cue-driven engagement with smartphone-related signals. Functionally, the MFG integrates top-down control with motivationally relevant inputs, modulating cognitive regulation of craving and impulsive tendencies [47, 48]. The observed thalamic activation in the PHONE > NEU contrast complements this interpretation, as thalamic nuclei are central relay structures facilitating information flow between prefrontal and sensory regions, thereby contributing to heightened salience attribution [49]. It is noteworthy that a very recent study reported sex differences in MFG in drug users, with dorsolateral prefrontal cortex activity being associated with cue-induced drug craving [51].

Beyond MFG activity, male participants demonstrated broader cortical recruitment, encompassing increased activity of postcentral, parietal and occipital cortices in the ON > OFF condition. The co-activation of parietal and occipital regions in the ON > OFF contrast further delineates the sensory and attentional dimensions of smartphone cue processing in males, as also observed by previous CR-studies. The right angular gyrus, a key parietal hub involved in attention reorientation and self-referential processing [52], may support the integration of external visual cues with internal cognitive states related to craving reappraisal [53]. The left postcentral gyrus activation indicates possible sensorimotor resonance given the active vs. inactive cue presentation, and occipital activity, particularly in the left middle and inferior occipital gyri, likely reflects enhanced visual processing of salient cues. Collectively, these findings suggest that in males, smartphone cues elicit a distributed network response dominated by prefrontal engagement, integrating sensory, attentional, and motivational components that have repeatedly associated with salience attribution, urge control and craving [53–55]. However, while eye-tracking is not routinely implemented in fMRI cue-reactivity paradigms, its absence represents a relevant limitation for the differentiated appraisal of posterior cortical activation differences. For instance, very recent work in individuals at high risk for gaming disorder has demonstrated that eye-movement measures can capture early, group-specific attentional biases, reflected in significant alterations in saccade frequency and attentional shifting [56]. Accordingly, in the absence of concurrent eye-tracking, it cannot be fully excluded that sex-related variations in visual attention or gaze behavior contributed, at least in part, to the observed CR-related occipital and parietal activation patterns.

The pattern of associations between SPAI dimensions and brain activation revealed both convergent and divergent mechanisms across sexes, pointing to sex-specific neural correlates of smartphone-related behavioral tendencies. In males, positive correlations between SPAI total, craving, and sleep interference scores and right MFG activation suggest that higher addiction-related tendencies are accompanied by greater recruitment of executive control regions. This may reflect increased engagement of prefrontal networks to regulate heightened motivational salience or craving elicited by salient cues [50, 51, 57]. Conversely, in females, SPAI total, daily life interference, and craving scores were negatively correlated with activation in the right angular gyrus, indicating that stronger PSU-related symptoms are associated with reduced parietal activation during cue exposure. This negative association may reflect attenuated attentional reallocation or diminished integration of self-referential and visuospatial processes when faced with salient smartphone stimuli. Two plausible mechanisms could underlie these sex differences. First, neurobiological factors, such as sex hormone modulation of prefrontal cortex function, may influence differential patterns of cue-related control and attention between males and females [51]. Second, psychosocial and behavioral factors such as differences in smartphone use motives, cognitive control strategies, and habitual engagement [13] may shape distinct neural adaptations to cue exposure, with males showing greater recruitment of top-down control responses compared with females. This pattern can be seen as complementary to epidemiological findings suggesting that females are slightly more prone to PSU [2, 58].

Placed within a broader literature on sex differences in reward processing, impulsivity, and emotion regulation the present sex-specific activation patterns align with broader frameworks describing differences in reward processing, impulsivity, and emotion regulation between males and females. Large-scale fMRI studies using gambling and reward paradigms have shown that reward-driven impulsivity is associated with greater lateral prefrontal engagement in men, whereas in women it is more strongly linked to right-hemispheric cingulate and parietal regions [59]. These dissociable neural profiles parallel our findings of male-dominant frontal engagement and female-specific parietal associations with PSU behavior, suggesting that smartphone cues may differentially tap into sex-dependent control versus salience mechanisms. Complementary evidence from emotion-processing studies further indicates that sex-specific affective traits modulate attentional and self-referential networks, with occipital and parietal regions showing stronger modulation in men, and frontal regions in women [60], underscoring the role of individual emotional traits in shaping CR. Finally, converging data from substance-related CR research, including cannabis use, demonstrate attenuated cue responses in women relative to men alongside sex-specific associations with craving and use severity, reinforcing the notion that cue-induced motivational and control processes are organized along partially distinct neurobiological pathways across sexes [61]. Such findings provide a broader theoretical context in which the observed frontoparietal (and possibly also occipital) engagement patterns during smartphone CR may reflect sex-specific balances between reward sensitivity, impulse regulation, and affective salience [62, 63] rather than domain-specific effects of smartphone use alone.

Eventually, cross-modal correlation analyses revealed broad, sex-independent associations between smartphone cue reactivity and multiple neurotransmitter and cellular systems, indicating the involvement of both excitatory and inhibitory mechanisms. This pattern supports the view that cortical projection neurons and inhibitory interneurons jointly shape cue-related neural responses through coordinated regulation of motivational and control processes. Overall, these findings align with evidence from addiction research suggesting that smartphone CR engages an integrated cognitive–reward network balancing excitation, inhibition, and motivational salience [3, 19, 20]. Simultaneously, subtle sex-dependent trends emerged in 5HT4 and MU maps, which showed subtle differences in female and male participants. Although these findings did not survive FDR-correction, they nonetheless point to possible sex-specific neurochemical modulation, particularly within serotonergic and opioidergic systems, both known to differ in receptor distribution and functional dynamics between sexes. Such differences may contribute to sex-related variability in affective and motivational responses to smartphone cues, consistent with prior work highlighting serotonergic and opioidergic involvement in adaptive, stress-related, and reward-regulatory processes [18, 64, 65]. Albeit not FDR-significant, the associations with glia-astrocyte maps are intriguing, as astrocytes are implicated in addiction and relapse, play key roles in axon guidance and modulation of neuronal activity, and thus contribute to the formation and weighting of neural connections [66, 67]. This finding, not previously suggested by existing data in IGD or PSU research on CR, may warrant further investigation in future studies exploring cellular contributions to addictive digital behaviors.

We acknowledge limitations of this study, including the relatively modest sample size, which may have limited the detection of subtler effects. In particular, the unequal sex distribution, with a smaller male subsample compared to females, may have reduced power for sex-comparative analyses and limits the generalizability of null findings, especially with respect to the absence of female-dominant activation patterns. As such, conclusions regarding the lack of stronger neural responses in females should be interpreted with appropriate caution. The cross-sectional design also precludes causal inferences, and the use of uncorrected thresholds in some contrasts warrants cautious interpretation of exploratory results, particularly with respect to associations between CR-derived brain activity, neurotransmitters and cellular markers. In this context, the breadth of PET/SPECT-derived neurochemical maps examined implies that these cross-modal patterns are best understood as exploratory signals intended to inform future targeted investigations rather than as definitive evidence of specific neurochemical mechanisms. Further, although the use of an uncorrected voxel-wise threshold combined with cluster-extent thresholding is a commonly accepted approach for exploratory analyses, these findings should be regarded as preliminary and require replication in larger samples using more stringent multiple-comparison correction procedures. Further, while the absence of spatial overlap supports the task-specific nature of the observed effects, a modulation of task-related regions by areas identified in the ALFF maps through underlying functional connectivity cannot be fully excluded. Eventually, we did not control for menstrual cycle phase or collect hormonal data in female participants. Given well-established effects of sex hormones on neural reactivity and neurotransmitter systems, hormonal variability may have contributed to within-group heterogeneity in females and potentially obscured sex-specific effects. Future studies incorporating hormonal assessments and cycle phase characterization are therefore warranted.

Keeping these limitations in mind, this study provides initial evidence that males show enhanced activation within frontoparietal cortices during processing of smartphone cues, accompanied by sex-divergent associations between neural responses and behavioral dimensions of smartphone use. However, given the imbalanced sample sizes and exploratory statistical thresholds, conclusions regarding the absence of female-dominant neural activation should be interpreted with caution. Cross-modal analyses further suggest that these processes engage both excitatory and inhibitory components across both females and males, indicating largely shared neurochemical substrates independent of sex. Notably, the identified sex-specific neural patterns may carry translational relevance by pointing to partially distinct neural targets for prevention and intervention, with greater emphasis on prefrontal control mechanisms in males and on attentional or self-referential processes in females. Overall, the data offer converging evidence for sex-related neural and behavioral patterns associated with maladaptive smartphone use and underscore the biological relevance of CR as a mechanistic marker of excessive engagement. Future research should extend these findings using larger, sex-balanced, and hormonally characterized samples, as well as longitudinal designs, to more precisely delineate sex-specific neural plasticity and neurochemical modulation in smartphone-related behaviors.

## Supplementary Information

Supplementary Material 1

## Data Availability

The data are available from the corresponding author upon reasonable scientific request.
