# Supplementary material for: Sex-specific neural responses to smartphone cues in young adults
Source: Biol Sex Differ. 2026 Jan 31;17:39. doi: 10.1186/s13293-026-00835-7 (PMC12930991; doi:10.1186/s13293-026-00835-7)
Supplement: Supplementary file 1 — Supplementary Material 1 [file 13293_2026_835_MOESM1_ESM.docx]

| **Table S1** Differences of resting-state brain activation between groups. | | | | | |
| --- | --- | --- | --- | --- | --- |
| Brain area | Cluster size k (voxel) | T value (peak voxel) | Peak voxel coordinates (MNI) | | |
|  |  |  | x | y | z |
| *ALFF male > female; k = 8* | | | | | |
| L inferior temporal gyrus | 657 | 5.54 | -36 | -60 | 0 |
| R putamen | 239 | 4.99 | 30 | 0 | 12 |
| L superior frontal gyrus | 50 | 4.98 | -27 | 15 | 66 |
| L opercular part of the inferior frontal gyrus | 21 | 4.90 | -60 | 12 | 3 |
| R fusiform gyrus | 47 | 4.50 | 42 | -45 | -9 |
| R thalamus proper | 23 | 4.32 | 12 | -6 | -6 |
| R anterior insula | 16 | 4.26 | 27 | 33 | -3 |
| L pallidum | 16 | 4.22 | -15 | -6 | -6 |
| L middle frontal gyrus | 12 | 4.04 | -39 | 60 | 9 |
| L inferior temporal gyrus | 19 | 4.03 | -33 | -3 | -39 |
| L fusiform gyrus | 15 | 3.97 | -30 | -24 | -39 |
| L parietal operculum | 8 | 3.95 | -36 | -45 | 21 |
| R anterior orbital gyrus | 9 | 3.83 | 30 | 42 | -21 |
| R thalamus proper | 28 | 3.73 | 27 | -24 | -3 |
| R occipital pole | 16 | 3.71 | 24 | -99 | 18 |
| L occipital pole | 9 | 3.67 | -12 | -99 | 24 |
| L superior occipital gyrus | 10 | 3.50 | -18 | -78 | 36 |
| *ALFF female > male; k = 8* | | | | | |
| R cerebellum exterior | 1494 | 6.53 | 33 | -69 | -54 |
| L posterior cingulate gyrus | 108 | 4.78 | 0 | -45 | 21 |
| R middle frontal gyrus | 46 | 4.24 | 27 | 18 | 42 |
| L superior frontal gyrus | 17 | 4.11 | -12 | 33 | 48 |
| Brain stem | 23 | 4.09 | 15 | -30 | -30 |
| R superior frontal gyrus medial segment | 10 | 3.94 | 9 | 45 | -9 |
| L medial frontal gyrus | 24 | 3.92 | -9 | 45 | -15 |
| Brain stem | 17 | 3.87 | -6 | -36 | -9 |
| L temporal pole | 17 | 3.85 | -39 | 3 | -21 |
| R precentral gyrus | 19 | 3.80 | 18 | -15 | 66 |
| L superior frontal gyrus | 18 | 3.71 | -12 | 3 | 63 |
| L postcentral gyrus | 10 | 3.62 | -45 | -33 | 54 |


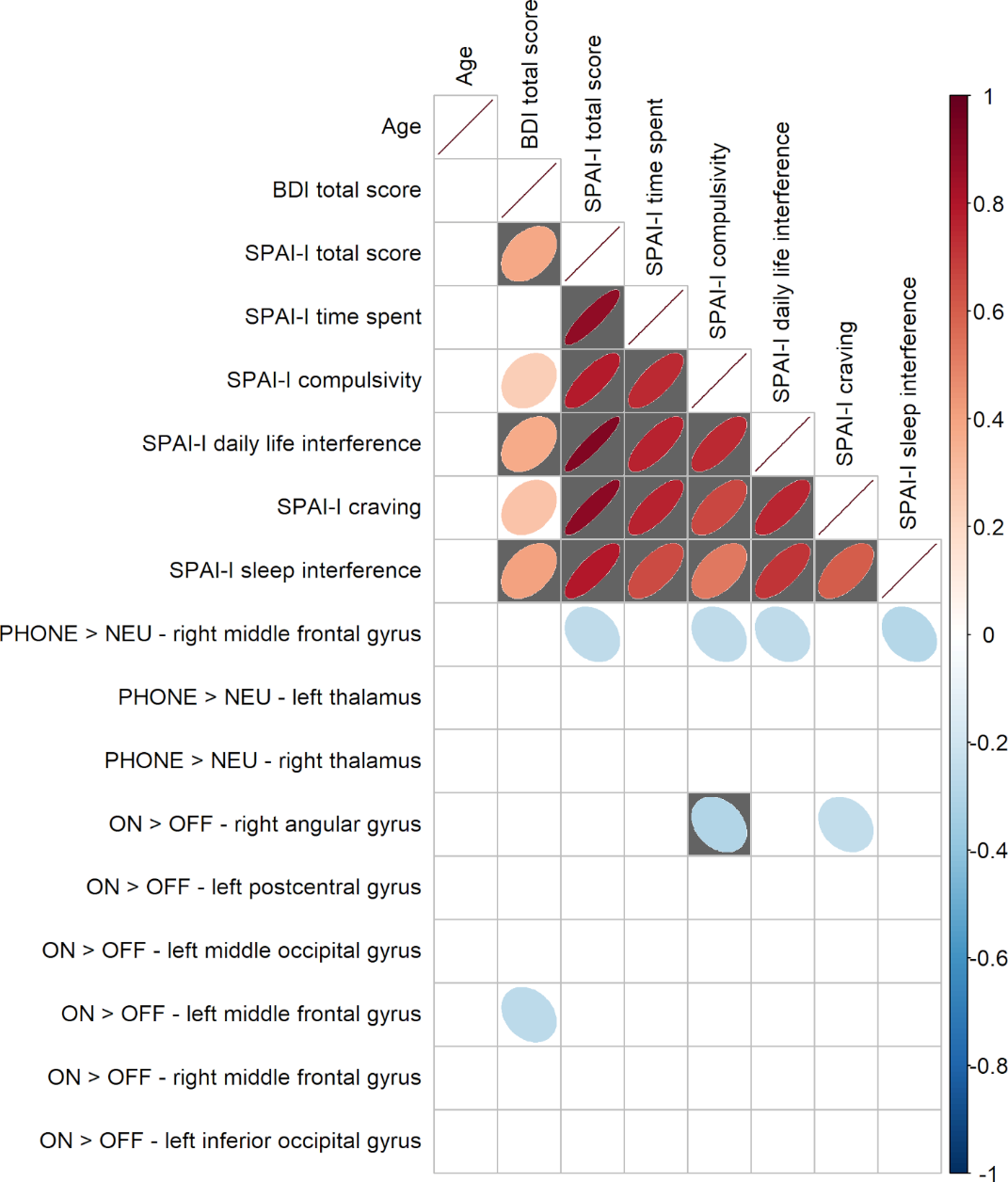


**Figure S1.** Correlation matrix of Spearman rank correlations between psychometric data and task-based brain activity within regions of interest based on group differences in female and male participants together.

Ellipses depict significant correlations at the *p* < 0.05 level, tests surviving FDR-correction are highlighted in gray color. This figure was created using R and GIMP.


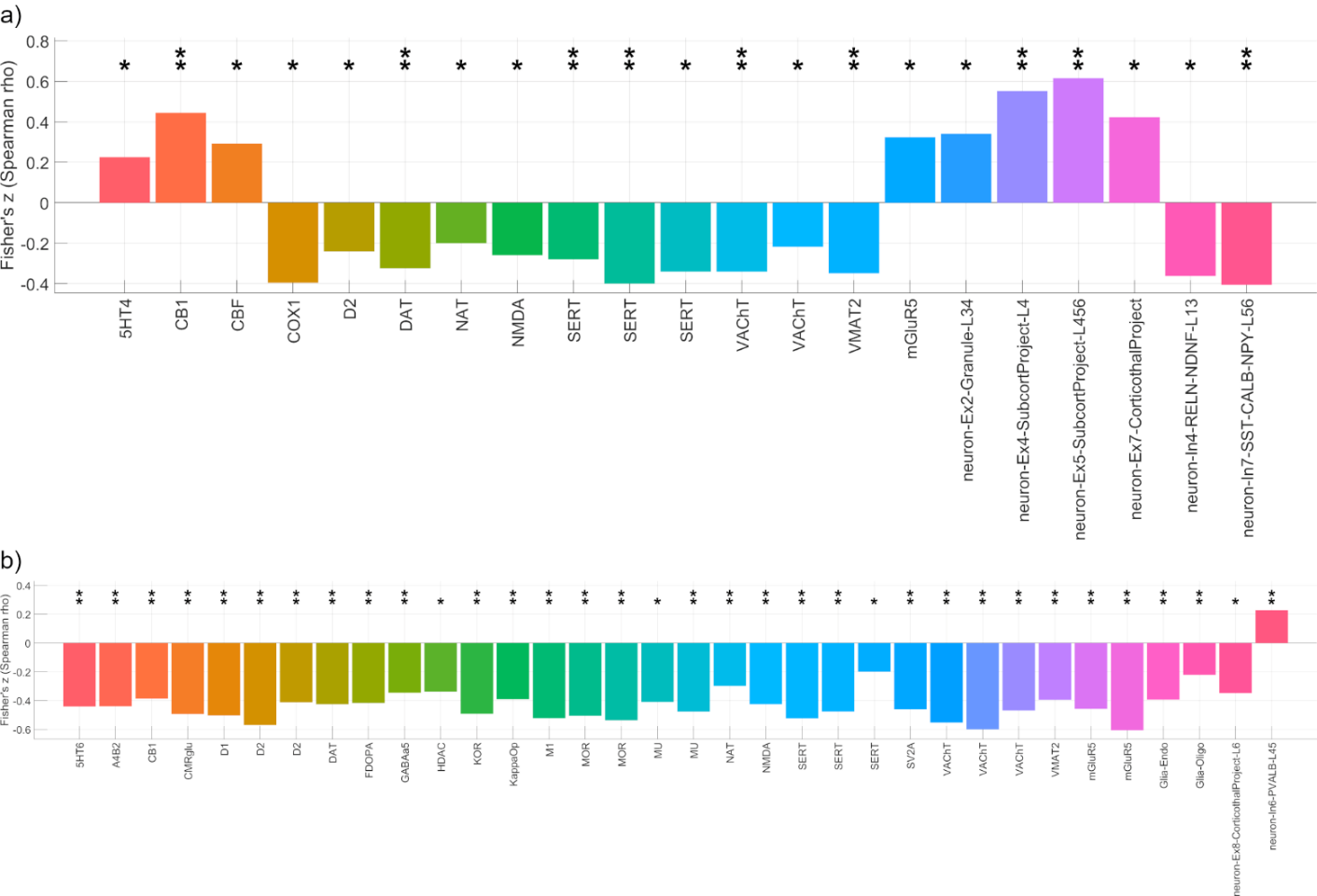


**Figure S2.** Cross-modal Spearman correlations between brain activation and receptor probability maps/cellular markers.

a) PHONE > NEU female and male together, * exact *p* < 0.05, ** survives FDR correction. b) ON > OFF female and male together, * exact *p* < 0.05, ** survives FDR correction. This figure was created using JuSpace and GIMP.


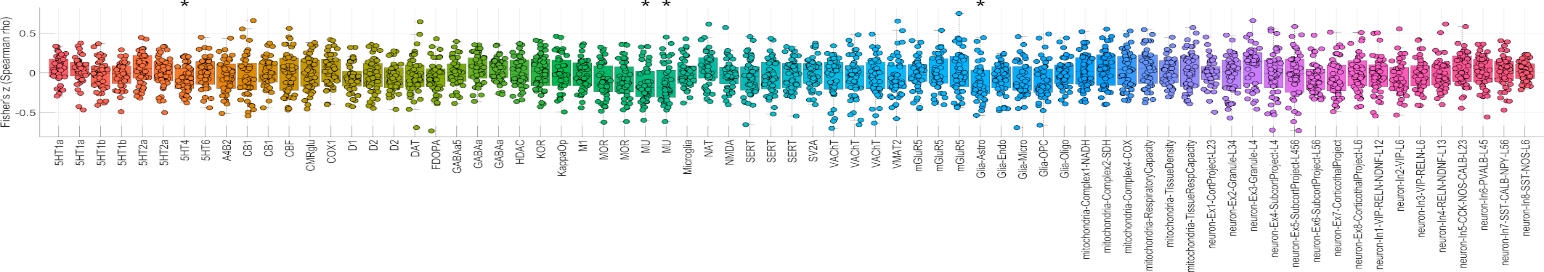


**Figure S3.** Differences of cross-modal Spearman correlations between brain activation and receptor probability maps/cellular markers (full analysis set).

PHONE > NEU female vs. male, * exact *p* < 0.05, none of the in-total 71 correlations survived FDR-correction. This figure was created using JuSpace and GIMP.


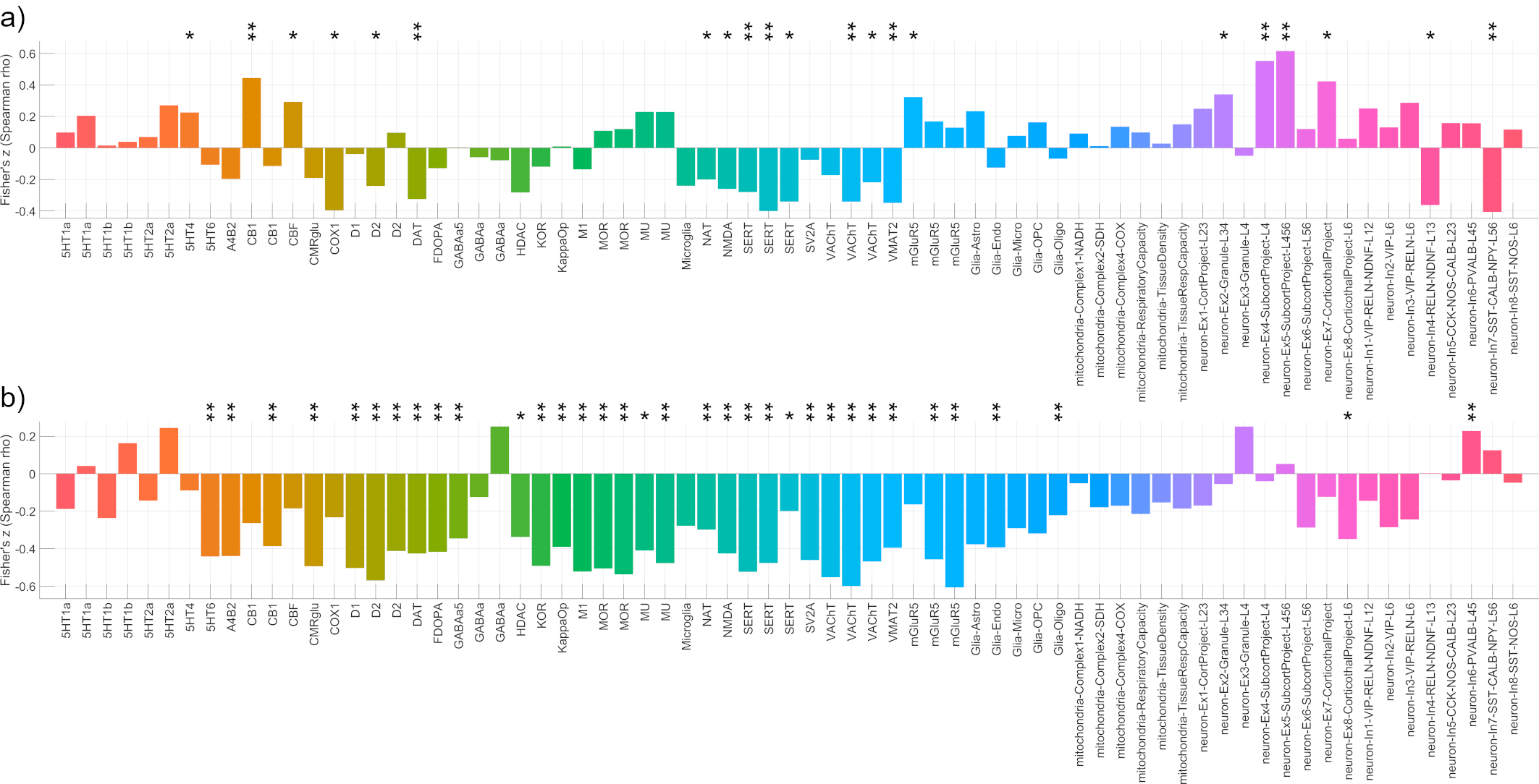


**Figure S4.** Cross-modal Spearman correlations between brain activation and receptor probability maps/cellular markers (full analysis set).

a) PHONE > NEU female and male together, * exact *p* < 0.05, ** survives FDR correction. b) ON > OFF female and male together, * exact *p* < 0.05, ** survives FDR correction. This figure was created using JuSpace and GIMP.
